# Upcycling Oat Hulls via Solid-State Fermentation Using Edible Filamentous Fungi: A Co-Culture Approach with *Neurospora intermedia* and *Rhizopus oryzae*

**DOI:** 10.3390/jof11110810

**Published:** 2025-11-14

**Authors:** Laura Georgiana Radulescu, Mikael Terp, Christian Enrico Rusbjerg-Weberskov, Niels Thomas Eriksen, Mette Lübeck

**Affiliations:** Department of Chemistry and Bioscience, Aalborg University, 9220 Aalborg, Denmark; radulesculaura6@gmail.com (L.G.R.); mter@bio.aau.dk (M.T.); cerw@bio.aau.dk (C.E.R.-W.); nte@bio.aau.dk (N.T.E.)

**Keywords:** solid-state fermentation, filamentous fungi, oat hulls, co-culture, *Neurospora intermedia*, *Rhizopus oryzae*, alternative proteins, waste valorization

## Abstract

The global challenge of food insecurity requires innovative approaches for sustainable food production and waste valorization. This study investigates the valorization of oat hulls, an abundant lignocellulosic by-product from oat manufacturing, by solid-state fermentation using edible filamentous fungi. Oat hulls sourced from oatmeal industrial side-streams were used as the sole substrate in co-cultures of *Neurospora intermedia* and *Rhizopus oryzae*. The fermentation process was optimized and upscaled, with fungal growth monitored via CO_2_ efflux and modeled to assess substrate utilization. Comprehensive analyses revealed a significant increase in protein concentration (*p* < 0.05) in the fermented oat hulls compared to the non-fermented controls. The resulting product was successfully incorporated into granola bars, which underwent sensory evaluation and received positive feedback, demonstrating its potential as a value-added food ingredient. These findings highlight the feasibility of using edible fungi to upcycle cereal processing by-products into nutritionally enhanced alternative protein sources, supporting both food system sustainability and circular bioeconomy objectives.

## 1. Introduction

Food security is a widely acknowledged global priority, as defined by the second Sustainable Development Goal of the United Nations, requiring access to sufficient, safe, and nutritious food that meets the dietary requirements of all people at all times [1,2]. Achieving this goal requires the building and refinement of food systems that are both sustainable and capable of meeting the dietary demands of a rapidly growing global population [3]. Tackling this major issue requires innovative strategies and transformative approaches to ensure equitable access to food and long-term resilience in food production systems [2,3]. One critical issue is the increasing demand for high-quality protein, which is contributed by livestock [4]. However, animal farming has a significant environmental impact [5], and meat consumption may be a health concern [6]. As a result, there is increasing interest in alternative protein sources (APs) [7], including those derived from plants, insects, and microorganisms [8]. However, scaling up AP production in a cost-effective and sustainable manner remains a significant hurdle, particularly because of the challenges of replicating the functional and nutritional properties of animal proteins [9].

Upcycling of organic side-streams has emerged as a sustainable alternative to traditional recycling [10]. Upcycling not only reduces food waste and environmental burden but also supports the circular bioeconomy by transforming low-value side-streams into high-quality food products [11,12]. Lignocellulosic biomass represents an abundant and underutilized resource with potential for valorization [13]. The recalcitrant nature of lignocellulose limits its direct use in food applications [14], necessitating innovative bioprocessing strategies [15].

Fungi have attracted considerable attention in recent times, being the catalysts in fungal biorefineries, due to their ability to utilize a wide range of biomasses as substrates, including agricultural and industrial residues [16,17]. Filamentous fungi, in particular, are highly effective in degrading complex plant materials due to their secretion of a broad spectrum of hydrolytic enzymes, such as cellulases, xylanases, laccase, lignin peroxidases, proteases, and amylases [18,19], which break down cellulose, hemicellulose, lignin, proteins, and starch into their respective monomers [20]. The biomass of filamentous fungi in itself serves as a potential source of fibers, vitamins, proteins, pigments, and enzymes [15]. Among these, several filamentous fungi are generally recognized as safe (GRAS), such as *Aspergillus oryzae*, *Aspergillus niger*, *Fusarium venenatum*, *Neurospora intermedia*, and *Rhizopus oryzae* [21]. The integration of fungal biorefineries into existing value chains, thus, represents a promising strategy for upcycling low-value by-product and advancing the circular bioeconomy [11].

*Neurospora intermedia* is a filamentous fungus commonly found in nature as a saprophytic organism, thriving on burned vegetation and contributing to the decomposition of charred plant matter [22,23]. On the Indonesian island of Java, *N. intermedia*, along with other *Neurospora* species, play a key role in fermenting plant-based food byproducts to produce red oncom, a traditional fermented food [24,25]. Mycoprotein produced from *N. intermedia* offers a complete amino acid profile and is rich in potassium, iron, and fiber, making it an appealing ingredient for innovative meat-alternative products [23,26].

*Rhizopus oryzae* is another filamentous fungus commonly found in nature, in different soil types, decaying plant matter, fruits, vegetables, and seeds [27]. *R. oryzae* is notably effective in producing organic acids, lactic acid and fumaric acid, and is also involved in the fermentation of traditional foods and drinks, including tempeh, black oncom, ragi, and certain alcoholic beverages [27]. More recently, *R. oryzae* has been proven to be a promising source of cellulolytic and xylanolytic enzymes, making it effective in converting agro-based lignocellulosic biomass, such as raw oil palm frond leaves, into valuable products [28]. In addition, fermentation of grains and beans by *R. oryzae* enriches flavor and increases the protein content of the substrate [29,30,31].

Agricultural by-products rich in lignocellulosic materials, such as oat hulls, wheat bran, and corn stover, are increasingly utilized as low-cost substrates for the production of fungal proteins [32]. Among these raw materials, *Avena sativa*, also commonly known as oat, has been cultivated worldwide for 2000 years [33]. Oats are among the most prominent cereal crops globally, ranking seventh in cereal production [34]. The processing of oats results in large amounts of oat hulls [35], as the hull comprise approximately 25% of the total mass of the seed [36]. The hulls consist mainly of the fibrous sheath that envelops the oat groat and lignocellulosic materials make up to 84% of the dry weight of oat hulls [37,38]. Oat hulls are approximately 23% cellulose, 35% hemicellulose, 25% lignin and 3% starch, the exact composition varying depending on growth conditions [37]. As a resource of lignocellulosic feedstock, oat hulls have previously been labeled as not having food value and are used primarily in the production of ethanol [39].

The use of co-culture in solid-state fermentation (SSF) introduces a novel approach by combining multiple fungal species to exploit the potential of synergistic interactions within metabolic pathways [40], leading to enhanced or cumulative effects such as increased yield of proteins or enzyme, activation of silent biosynthetic gene clusters, and increased production of secondary metabolites [41]. This strategy can be used in the process of lignocellulosic degradation to better break down plant cell walls, by creating a richer and more effective mixture of enzymes [42], or to generate novel metabolites that do not appear in monocultures [43]. Fungi are especially suitable for use in SSF, which closely resembles their natural habitat [44]. By leveraging the metabolic diversity and cooperative dynamics of co-cultured fungi, this strategy offers great potential for upcycling low-value biomass into high-value food ingredients, representing a significant advancement in the field of fungal biorefineries.

In this study, the valorization of oat hulls from oat production using a co-culture of the edible filamentous fungi, *N. intermedia* and *R. oryzae*, in SSF was investigated. The main aim of this study is to develop a sustainable process to valorize oat hulls into a nutritionally enhanced food ingredient. The findings support the potential of fungal bioprocessing as a key strategy for sustainable protein production and food waste upcycling, serving as proof-of-concept for the potential in utilizing co-cultures of synergistic filamentous fungi.

## 2. Materials and Methods

### 2.1. Compatability Assay

*Neurospora intermedia* (NRRL 2884) [45] and *Rhizopus oryzae* (CCUG 61147) were simultaneously cultivated at opposing edges of 2% agar (VWR International, Søborg, Denmark) plates containing 5% (*w*/*v*) milled oat hulls (Good Food Group, Vejle, Denmark) (autoclaved at 121 °C, 15 min), by scraping hyphae from culture plates, and incubating the co-culture for 72 h at 28 °C. The compatibility of the two species was evaluated macroscopically and under 10× magnification to facilitate detailed examination of growth interactions or zone demarcations.

### 2.2. Substrate Composition Optimization

Small-scale SSF was performed in Petri dishes (90 mm) (Hounisen Lab Equipment, Skanderborg, Denmark) containing different compositions of milled and whole hulls. The effect of substrate composition was investigated by preparing two media differing in the ratio of milled to whole oat hulls (1:1 and 2:1, both at 60% moisture content). The media were autoclaved in large containers (121 °C, 15 min), cooled to ambient temperature (23–25 °C) and distributed in Petri dishes. Both media were inoculated with co-cultures (*N. intermedia* $2.0\times 10^{4}$ conidia g^−1^ wet media and *R. oryzae* $2.4\times 10^{4}$ spores g^−1^ wet media). Cultures were incubated for five days at 28 °C. The optimal substrate composition was chosen based on the elemental analysis results showing the highest protein concentration.

### 2.3. Moisture Level Optimization

The effect of moisture level was studied using a substrate composition of 1:1 milled to whole hulls adjusted to moisture levels of 50%, 60%, and 70%. The substrates were first sterilized by autoclaving (121 °C, 15 min), cooled to ambient temperature, and distributed in Petri dishes. The inoculation with the co-culture was done as described above, and the plates were incubated for six days at 28 °C. The optimal amount of moisture was determined based on dry matter (DM) loss, by transferring the entire content of each plate of fermented oat hulls to pre-weighted aluminum trays, and oven dried (105 °C, for 24 h) until constant weight (maximum deviation of ±0.1%). The difference between the initial mass of dry matter and the mass of dry matter after fermentation was used to calculate the DM loss. Analysis of variance (ANOVA) was used to compare the effect of different moisture contents. Statistical analysis was performed using R version 4.3.1 and RStudio 2024.12.1 + 563.

### 2.4. Temperature Optimization

The optimal fermentation temperature was determined in chambers consisting of 3 L plastic containers (17 × 17 cm) with filter-fitted lids (SacO2, Deinze, Belgium), this being the upscaling from Petri dishes. Each box contained media composed of 75 g milled oat hulls and 75 g whole oat hulls with moisture content of 60%. The boxes were sterilized at 121 °C for 15 min and inoculated with the co-culture as described above. The fermentations were carried out for six days in incubators at 20 °C and 28 °C and evaluated by measuring the increase in protein concentration between non-fermented oat hulls and fermented ones, using elemental analysis.

### 2.5. Elemental Analysis for Protein Measurement

Elemental analysis was performed on 3–4 mg of sample material that was analyzed using Flash SMART^*TM*^ Elemental Analyzer (Thermo Scientific, Waltham, MA, USA) in CHN mode. Acetanilide (OEA labs, Exeter, UK) was used for calibration as reference standard. The combustion reactor temperature was 950 °C, the run time per sample was 420 s, helium carrier flow of 140 mL mn^−1^, reference flow of 100 mL mn^−1^, and oxygen flow of 250 mL mn^−1^. The carbon to nitrogen (C/N) ratio was determined on a DM basis using the area under the peak signal and the calibration curve constructed from the %C and %N content of the acetanilide standards. Nitrogen content was converted to crude protein concentration using a conversion factor of 6.25.

### 2.6. CO_2_ Evolution Rate Measurement

CO_2_ evolution rate (CER) was measured in order to predict the growth process and the biomass accumulation, of *N. intermedia* and *R. oryzae* co-culture during the fermentation of oat hulls [46]. CER was also measured for monocultures of *N. intermedia* and *R. oryzae*, respectively. The setup included a power supply, timer, air pump (SuperFish Air Flow, Avifauna Grenaa, Trustrup, Denmark), water tank for humidification, air filter (Frisenette Q-Max^*TM*^ filter, Mikrolab Aarhus, Viby J, Denmark), 12 L cylindrical fermentation chamber (24 d × 26.5 h cm), CO_2_ sensor (PASCO Scientific^TM^ Wireless CO_2_ Sensor, Roseville, CA, USA), and an outlet for exhaust air. The substrate composed of 400 g 1:1 milled to whole oat hulls wet media with 60% moisture, was incubated at 28 °C for 6 days in the fermentation chamber. The cultures were periodically aerated for periods of 15 min followed by periods of 45 min in which the fermentation chamber was closed [47]. CO_2_ concentration measurements were collected every minute using SPARKvue (PASCO Scientific^*TM*^, Roseville, CA, USA). During the periods in which the fermentation chamber was closed, CER was evaluated from the linear increase in CO_2_ concentration. We assumed the same CER for the following 15 min period when the aeration was on. After fermentation, samples were taken from the fermentation chamber and analyzed by SDS-PAGE to visualize differences in protein profiles between monocultures and co-culture.

### 2.7. Fungal Growth Modeling

The hourly rate of CO_2_ evolution was used for the modeling of the CO_2_ evolution over 120 h of fermentation. The Luedeking–Piret model was used as the base to predict the CER as a function of fungal biomass [48]:

(1) CER=(αμ+β)X

where CER is the CO_2_ evolution rate (mol h^−1^), α is the yield coefficient, μ is the specific growth rate (h^−1^), β is the maintenance coefficient, and X is the fungal biomass (g).

The final model was based on the Luedeking–Piret model from Equation (1) combined with Monod kinetics accounting for the fluctuations in CER by introducing five different substrates $S_{1}$–$S_{5}$ [49]. A detailed description of the model is shown in Supplementary Information S2. In brief, the specific growth rate $\mu_{i}$ on substrate $S_{i}$ is calculated as shown in Equation (2).

(2) $$\mu_{i}=\frac{\mu_{max,i}\times S_{i}}{K_{S,i}+S_{i}}$$

where $\mu_{max}$ is the maximal specific growth rate (h^−1^) on substrate $S_{i}$, $S_{1}$ is the amount of available substrate (g), and $K_{S,i}$ is the half saturation constant (g) on substrate $S_{i}$.

To capture the dynamics of the fluctuating CER, the model differentiated growth on the 5 substrate, $S_{1}$–$S_{5}$, which were assumed to be of variable quality and consumed in a sequence that resulted in stepwise decreasing specific growth rates. The fungal biomass, X, was calculated numerically while also taking maintenance, in terms of carbon equivalents, into account.

(3) $$\begin{array}{c} X_{t+\Delta t}=X_{t}+Y_{X/S1}\left(S_{1,t+\Delta t}-S_{1,t}\right)+Y_{X/S2}\left(S_{2,t+\Delta t}-S_{2,t}\right)+Y_{X/S3}\left(S_{3,t+\Delta t}-S_{3,t}\right)\\ +Y_{X/S4}(S_{4,t+\Delta t}+Y_{X/S5}\left(S_{5,t+\Delta t}-S_{5,t}\right)-\beta X_{t}\Delta t \end{array}$$

where $X_{t+\Delta t}$ is the biomass at the next time step and $X_{t}$ is the current biomass concentration. The terms of the form $\left(S_{i,t+\Delta t}-S_{i,t}\right)$ represent the change in substrate *i* over the time interval Δt. The yield coefficients $Y_{X/Si}$ convert these substrate changes into the corresponding biomass production in terms of carbon equivalents. $\beta X_{t}\Delta t$ accounts for the maintenance energy requirements of biomass and represents the portion of biomass that is lost during the time interval.

Substituting the Equations (2) and (3) into the Luedeking–Piret model from Equation (1), the model now takes into account the utilization of five substrates.

(4) $$\begin{array}{c} CER\left(t\right)=\left(\alpha_{1}\mu_{1}+\alpha_{2}\mu_{2}+\alpha_{3}\mu_{3}+\alpha_{4}\mu_{4}+\alpha_{5}\mu_{5}\right)X_{t}+\beta X_{t}\\ \;\;\;\;\;\;\;=\left(\left(1-Y_{X/S1}\right)\mu 1+\left(1-Y_{X/S2}\right)\mu 2+\left(1-Y_{X/S3}\right)\mu 3+\left(1-Y_{X/S4}\right)\mu 4+\left(1-Y_{X/S5}\right)\mu 5+\beta \right)X_{t} \end{array}$$

Equation (4) sums the contributions from each substrate, weighted by their respective specific growth rates and coefficients. $\alpha_{1}\mu_{1}+\alpha_{2}\mu_{2}+\alpha_{3}\mu_{3}+\alpha_{4}\mu_{4}+\alpha_{5}\mu_{5}$ represent CER’s directly from growth on each substrate. $\beta X_{t}$ represents maintenance-related CO_2_ production. $\alpha_{i}=\left(1-Y_{X/Si}\right)$, where $Y_{X/Si}$ represents how efficiently substrate carbon is converted to biomass carbon, which means that $\left(1-Y_{X/Si}\right)$ represents the fraction of carbon that is not incorporated into biomass and is instead released as CO_2_.

The modeling was used to predict CER from the co-culture of *N. intermedia* and *R. oryzae*, as well as for the monocultures of both fungal species.

### 2.8. SDS-PAGE Analysis

Samples were analyzed by SDS-PAGE to evaluate their protein profiles. Proteins were extracted from 6 mg milled sample by adding 200 μL reducing SDS sample buffer (25 mM Tris-HCl pH 6.8, 1% SDS, 20% glycerol, 25 mM dithiotreitol, trace of bromophenol blue (Thermo Scientific, Waltham, MA, USA)), vortexing, and incubating at 95 °C for 10 min followed by centrifugation to recover supernatant. A volume of 10 μL was loaded into the wells of 4–20% Bis-Tris SUREpage gel together with the protein ladder 6 μL Pierce Unstained Protein MW Marker # 26610 (Thermo Scientific, Waltham, MA, USA). The gel was stained with InstantStain Comassie blue (Kem-En-Tek Nordic A/S, Taastrup, Denmark). Picture of the SDS-PAGE gel was taken using a BioRad ChemiDocTM MP Imaging System (Bio-Rad, Hercules, CA, USA).

### 2.9. Sugar Identification

Two step acid hydrolysis was performed to determine the percentage of lignocellulose degradation after the fermentation [50,51]. Sulfuric acid (VWR International, Søborg, Denmark), 3 mL of 72%, was added to 0.3 g of sample material and incubated at 30 °C with 150 rpm for 60 min. Sugar recovery standards (SRS) (glucose, xylose, arabinose, and galactose (Thermo Scientific, Waltham, MA, USA)) were used to correct for losses due to destruction of sugars. The sulfuric acid concentration was diluted to 4% using ELGA water (Veolia Water Technologies, High Wycombe, UK). The tubes were vortexed and autoclaved at 121 °C for 60 min. The solid and liquid fractions were separated by centrifugation (8000× *g*). A total of 1 mL of the liquid fraction was filtered through a 0.22 μm filter into glass vials for HPLC (Waters, Milford, MA, USA) analysis of sugars. From each sample, 10 μL were led through the column (300×7.8 mm Rezex*^TM^* ROA-Organic Acid H+ (8%) LC Column (Phenomenex, Torrance, CA, USA)) using a mobile phase consisting of 5 mM sulfuric acid with a flow rate of 0.6 mL/min. The samples were run for 25 min to detect the sugars using the Waters 2414 Refractive Index Detector (Waters, Milford, MA, USA). The rest of the aliquot was used to quantify the amount of soluble lignin by using UV-vis spectrophotometry (240 nm), using sulfuric acid (4%) as control. The solid faction was used to determine the acid insoluble lignin, calculated as the difference between the dry mass of the acid insoluble residue and ash content of the sample on a DM basis [51].

### 2.10. Product Development

Fermented oat hulls were oven dried at 60 °C for 72 h and milled into a coarse or fine powder with 1 mm and 0.5 mm particle size, respectively. Varying inclusion rates (IR) of fermented oat hulls (0%, 15%, 20%, 25%) were tested to replace rolled oats in granola bars. The final granola bars consisted of 22.22% binding syrup (peanut butter and honey) and 77.78% dry matter (oats, fermented oat hulls, almonds, dates). A non-blind survey based on appearance, smell, texture, taste, and overall acceptability was conducted on 17 untrained participants to obtain a general evaluation of the prototype.

## 3. Results

### 3.1. Fermentation Variables Optimization

**Figure 1:**
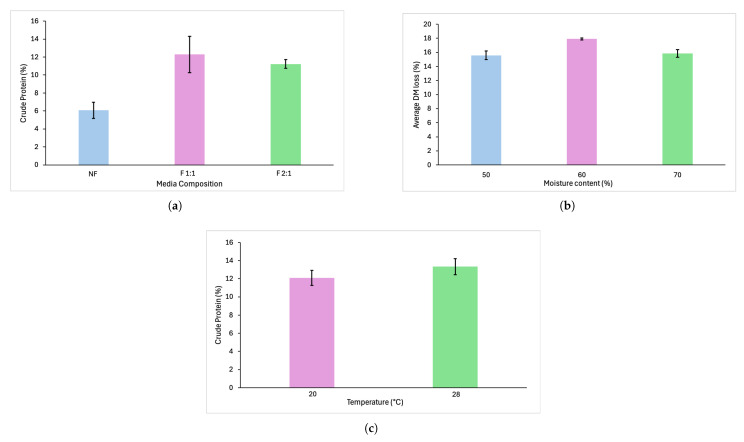
(**a**) Average crude protein concentration with standard deviations based on elemental analysis for the different media composition: NF—non-fermented oat hulls; F 1:1—fermented oat hulls with the co-culture of *N. intermedia* and *R. oryzae* on the 1:1 milled to whole hulls media; F 2:1—fermented oat hulls with the co-culture of *N. intermedia* and *R. oryzae* on the 2:1 milled to whole hulls media. (**b**) DM loss measured at 50%, 60%, and 70% moisture content of 1:1 oat hulls fermented with the co-culture of *N. intermedia* and *R. oryzae*. (**c**) Crude protein concentration of fermented oat hulls estimated after SSF using co-cultures of *N. intermedia* and *R. oryzae* at two different temperatures: 20 °C and 28 °C.

The optimal parameters for SSF to support protein production were 1:1 milled to whole hulls ratio with 60% moisture content, incubated at 28 °C. First, the milled to whole hulls ratio was tested for the best medium composition. The two different media inoculated with the co-culture were compared based on the crude protein concentration. Non-fermented oat hulls showed low protein concentration (5.45–6.72%) (Figure 1) compared to the fermented hulls with the co-culture of *N. intermedia* and *R. oryzae* (10.87–13.73%). The 1:1 ratio of milled to whole hulls had the highest crude protein concentration based on the results of the elemental analysis (13.73%) and was used for further experiments.

The optimal humidity content was determined based on the highest DM loss. It occurred consistently at 60% moisture content, with values approximately 2% higher than the 50% and 70% moisture content (Figure 1). Based on the two-factor ANOVA and Tukey test performed on DM loss, the difference between the 60% moisture content was statistically significant (*p* < 0.05) from the other two moisture contents and was used for the further experiments.

The temperature with the highest average crude protein concentration after fermentation was determined to be at 28 °C (13.33%) compared to 20 °C (12.10%), in the upscaled bioreactors (375 g of moist media on 289 cm^2^ surface area).

### 3.2. Growth Modeling

**Figure 2:**
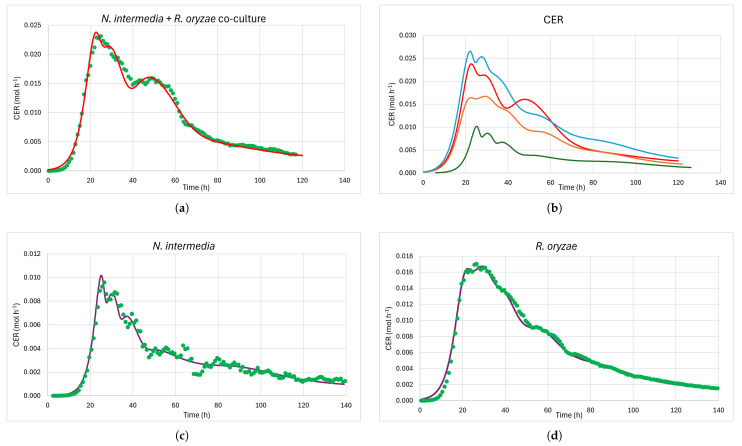
(**a**) Measured (green dots) and modeled (red curve) CER from *N. intermedia* and *R. oryzae* co-culture. (**b**) Comparison of model data: co-culture of *R. oryzae* and *N. intermedia* (red); *R. oryzae* monoculture (orange); *N. intermedia* monoculture (green); sum of CO_2_ production rates of both monocultures (light blue). (**c**) Measured (green dots) and modeled (purple curve) CER from *N. intermedia* monoculture. (**d**) Measured (green dots) and modeled (purple curve) CER from *R. oryzae* monoculture.

Carbon dioxide production is linked to microbial activity, therefore, an indirect estimation of fungal growth kinetics was made based on CO_2_ efflux measurements [52]. Using the predicted values of specific growth rates and substrate consumption of five different substrates ($S_{1},S_{2},S_{3},S_{4},S_{5}$), the final model for CER was obtained by integrating Monod and Luedeking–Piret kinetics. The predictive power of the model was evaluated by comparing its output with the experimental data. The model captured the main peaks and declines in CER. The co-culture (Figure 2a) had high double peak between 20 and 30 h, presumably corresponding to highest specific growth rates ($\mu_{1},\mu_{2}$) of the first two substrates ($S_{1},S_{2}$) that are consumed sequentially. The first major decline in CER between 30 and 40 h of fermentation, and the second major peak with lower rates of CER between 40 and 60 h, correspond to the third and fourth substrates consumed ($S_{3},S_{4}$). $S_{5}$ started being consumed after 80 h of fermentation. The fermentation was ended on Day 5 days when the CO_2_ production rate had fallen to 10–15% of the maximal rate detected earlier in the process, indicating that most of the degradable carbon components had then been metabolized. The co-culture of *N. intermedia* and *R. oryzae* (Figure 2b) produced more CO_2_ and at a higher rate than any of the monocultures by themselves, almost as much as the sum of their individual CO_2_ production rates.

In monocultures of *N. intermedia* and *R. oryzae* (Figure 2c,d), the timing and magnitude of the CER peaks were well represented by the Model (Equation (4)). *N. intermedia* had a sharp peak with a maximum CER value of 0.01 mol h^−1^. A total of 0.39 mol of CO_2_, which corresponded to 17.2 g of CO_2_ (4.7 g of carbon), were lost during the fermentation of *N. intermedia*, see Supplementary Information S1. *R. oryzae* exhibited a double peak, with a maximum CER value of 0.017 mol h^−1^. In total, 0.84 mol of CO_2_, which corresponded to 37 g of CO_2_ (10.1 g of carbon), were lost during the fermentation of *R. oryzae*. After these peaks, both cultures showed a gradual decline in CER.

The co-culture of *N. intermedia* and *R. oryzae* had the largest amount of substrate lost as CO_2_, 1.06 mol of CO_2_ which corresponded to 46.4 g of CO_2_ (12.7 g of carbon), and the maximum CER of 0.023 mol h^−1^.

### 3.3. Protein Profile

**Figure 3:**
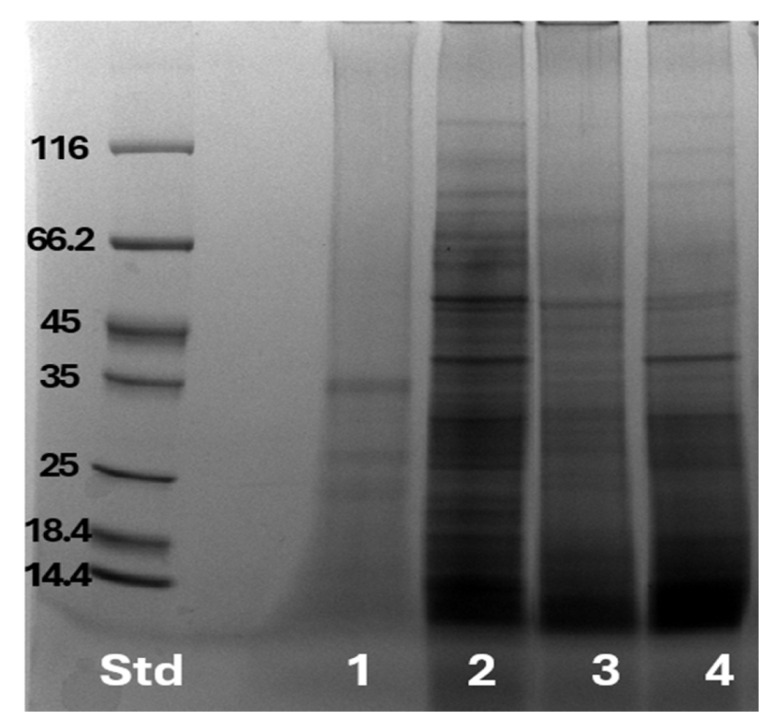
SDS-page analysis of protein composition in fermented and non-fermented samples. 1—non-fermented oat hulls; 2—*N. intermedia* monoculture; 3—*R. oryzae* monoculture; 4—co-culture of *N. intermedia* and *R. oryzae*.

Protein profiles from non-fermented oat hulls and oat hulls fermented in mono- or co-cultures were analyzed and compared using SDS-PAGE. In Figure 3 the non-fermented oat hulls showed protein bands in the 25–35 kDa range, representing the native protein content of oat hulls before fungal cultivation. Fermented oat hulls with *N. intermedia* monoculture lane displayed numerous protein bands across a wide molecular weight range, with particularly dense banding in the 14–35 kDa region. The *R. oryzae* monoculture lane also showed multiple protein bands, with a pattern distinct from *N. intermedia*. The co-culture of *N. intermedia* and *R. oryzae* exhibited a protein profile that appeared to incorporate elements from both monocultures and some bands appeared more intense than in either of the monocultures alone. The 35 kDa band observed in the non-fermented oat hulls was absent in the fermented sample, indicating proteolytic degradation of the hull proteins during fermentation. The SDS-PAGE analysis shows how fermentation changes the protein composition in the product to contain primarily mycoproteins.

### 3.4. Lignocellulose Degradation

Acid hydrolysis was used to quantify the monomeric sugars in both fermented and non-fermented oat hulls. The fermentation significantly reduced the glucan content from 36.31% to 22.85%. The xylan content remained relatively stable, from 19.67% to 20.91%, and arabinan decreased by roughly half from 6.40% to 3.13%. Acid insoluble lignin decreased slightly from 27.95% to 26.22%, while acid soluble lignin had a small increase in concentration. The ash content did not show significant change (Table 1).

### 3.5. Food Ingredient Prototype

**Figure 4:**
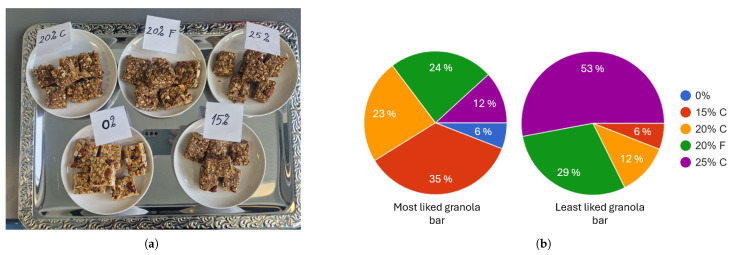
(**a**) Granola bars made with increasing inclusion rates (IR) of fermented oat hulls: 0%, 15% coarse milled (C), 20% C, 20% fine milled (F) and 25% C (**b**) Survey results of the preferences towards the different granola bar recipes (*n* = 17). 0%—standard granola bar without fermented oat hulls; 15% C—granola bar with 15% IR of fermented oat hulls coarse milled; 20% C—granola bar with 20% IR of fermented oat hulls coarse milled; 20% F—granola bar with 20% IR of fermented oat hulls fine milled; 25% C—granola bar with 25% IR of fermented oat hulls coarse milled.

Five batches of granola bars were made using peanut butter, honey, dates, roasted almonds, and oats (Figure 4a). Oat hulls fermented with the co-culture of *N. intermedia* and *R. oryzae* were used to replace the oats in the granola bars in increasing amounts. The granola bars had visible differences in appearance, the ones with higher inclusion rate (IR) having a darker color. The general comments include that all the bars had a neutral smell, 20% fine milled (F) was sweeter, while 15% coarse milled (C), 20% C, and 25% C were more dry. Despite the high rating on key parameters for IR 0%, the majority of the panel liked the 15% C best among all granola bars. The granola bar most of the participants liked least was the IR 25% C (Figure 4b), due to the unpleasant texture given by the higher amount of fibers, while also being too dry and crumbling compared to the other bars.

## 4. Discussion

In this study we successfully identified parameters to support SSF of oat hulls using a co-culture of *N. intermedia* and *R. oryzae*. Fermentation with the co-culture resulted in higher crude protein concentration on the 1:1 milled to whole hulls when compared to the protein concentration of the fermented 2:1 media. Milling increased surface area and facilitated faster nutrient access, yet both fungi colonized the hulls efficiently, suggesting robust enzymatic capabilities and structural adaptation. *N. intermedia* is recognized for its lignocellulolytic enzymes, whereas the filaments of *R. oryzae* may aid structural colonization. Together, these traits enabled efficient nutrient extraction even from intact hulls [53]. The lignocellulose degradation analysis proved that the differences between fermented and non-fermented oat hulls were statistically significant and that the observed changes were due to the fermentation process rather than to random variation. The reduction in glucan indicated effective cellulose (or starch) degradation by the fungal co-culture, meaning *N. intermedia* and *R. oryzae* mainly consumed glucose polymers during fermentation [25,54,55]. The slight increase in the xylan content could be due to microbes producing xylanases that modified the structure of xylan, making it more accessible for acid hydrolysis, or it could be due to the utilization of other components, which would relatively increase the percentage of xylan [56,57,58]. The slight decrease in acid insoluble lignin and the increase in acid soluble lignin indicate that the fermentation process partially converted insoluble lignin into more soluble forms, consistent with the limited ligninolytic capacity of *Neurospora crassa* [59]. However, the changes in both lignin fractions were small, suggesting that the co-culture had just a limited impact on lignin [60].

Moisture content strongly influenced the performance of the co-culture, reflecting its central role in SSF [61]. The 60% condition resulted in the highest DM loss, indicating optimal substrate utilization. At 50%, moisture limitation restricted enzymatic activity despite visible sporulation, while 70% moisture hindered oxygen transfer, limiting aerobic metabolism. These results clearly demonstrate that moisture content is a key variable in optimizing SSF [62,63].

Scaling experiments underscored the importance of process design in transitioning from laboratory to industrial dimensions, which would be relevant for the conversion of large amounts of oat hulls for the food industry. Petri dish systems provided valuable initial insights but did not capture heat transfer constraints typical for larger systems. As fungal metabolism is exothermic, heat buildup often limits SSF scalability [64,65]. In the present case, however, the intermediate scale (375 g of substrate on 289 cm^2^ surface) at 28 °C supported increased protein concentration without heat build-up that would result in suboptimal fermentation conditions for the fungi. This result strongly suggests that the chosen fungal consortium not only tolerates, but thrives under conditions approaching industrial relevance, further supporting its biotechnological potential.

Indirect estimation of biomass growth through CER measurement exploits the metabolic link between microbial activity and carbon dioxide production. This method is widely used in fermentation and fungal growth studies due to its non-invasive nature and real-time monitoring capabilities [66,67]. Differences in the CO_2_ evolution profiles (Figure 2) as well as the total amounts of CO_2_ produced (Supplementary Information, S1) suggested that substrates were utilized at variable rates and to varying extents during the fermentations. When comparing the performance of the co-culture of *N. intermedia* and *R. oryzae* with the monocultures, the co-culture CER exceeded the monoculture peaks (Figure 2b), suggesting synergistic metabolic interactions or additive substrate utilization. The initial peak could be attributed to both species utilizing preferred substrates, then a transition period as preferred substrates deplete, while the subsequent peaks represent consumption and growth on secondary substrates by one or both species. Based on the amount of substrate lost as CO_2_ it was revealed that the co-culture created a more productive metabolic system than monocultures alone, demonstrating better resource utilization that emerged from fungal interactions. Synergistic interactions in fungal co-cultures depend on specific physiological and metabolic compatibilities and are not invariably obtained by pairing different fungal species [68,69]. In this study, the combination of *N. intermedia* and *R. oryzae* yielded a productive, metabolically complex co-culture with enhanced resource utilization, demonstrated by elevated CER peaks and higher substrate conversion. The success of the *N. intermedia* and *R. oryzae* pairing reflects unique metabolic compatibilities, a finding echoed in other microbial co-culture studies where synergy is context- and species-dependent [69,70].

The model (Equations (1)–(4)) was able to accurately depict CER from the oat hull fermentation, irrespective of which fungi were employed, as long as it considered utilization of 5 distinct substrate classes, taken up sequentially in the order by which they were preferred by the fungi. This robust performance was achieved despite the absence of explicit knowledge about the specific substrates consumed and without including enzymatic mobilization of polymers into monomers from the oat hulls [49]. Future studies should also focus on the enzymes produced and the substrates utilized during the process. In this study, the experimental setup did not allow us to remove substrate samples without disturbing the CO_2_ measurements.

Regarding granola bars with fermented oat hulls they received a general positive response, with the majority of participants in the survey preferring those with fermented oat hulls to the regular granola bar. However, the opinions were very polarized when it came to texture and overall favorability due to personal preferences, some preferring a more soft texture (as was the case for IR 20% F) and others a crunchier texture (IR 15% C or IR 20% C). Considering that by including fermented oat hulls in granola bars, the fiber concentration increased, which could be an aspect of interest for consumers. Through fermentation, the protein concentration of the fermented oat hulls increased, making them comparable to rolled oats that are commonly used in granola bars, while having a higher fiber content [71].

## 5. Conclusions

We showed that by co-culturing, *N. intermedia* and *R. oryzae* can increase the protein concentration of oat hulls through SSF. The observed synergy results from more efficient carbohydrate degradation by the co-culture, as supported by the increased CER compared to monocultures. Furthermore, the fermentation process successfully upcycled oat hulls into a novel food ingredient, and the resulting granola bars were well received by the test panel. This study demonstrates a way to convert a lignocellulosic-rich by-product, oat hulls, into a novel food.

## Figures and Tables

**Table 1:** Sugar composition of the fermented oat hulls with the co-culture of *N. intermedia* and *R. oryzae*, and of the non-fermented oat hulls.

Compounds	Compounds in Fermented Oat Hulls (%)	Compounds in Non-Fermented Oat Hulls (%)
Glucan *	22.85 ± 0.38	36.31 ± 0.15
Xylan	20.91 ± 0.22	19.67 ± 0.83
Arabinan *	3.13± 0.03	6.40 ± 0.04
AIL ^1^	26.22 ± 0.02	27.95 ± 0.04
ASL ^2^	1.24 ± 0.04	1.14 ± 0.05
Ash	1.57 ± 0.30	0.85 ± 0.21

^1^ AIL—acid insoluble lignin; ^2^ ASL—acid soluble lignin; *—Statistically significant difference of the compound concentration between the fermented and non-fermented oat hulls, based on ANOVA.

## Data Availability

The original contributions presented in this study are included in the article/Supplementary Material. Further inquiries can be directed to the corresponding author.

## Supplementary Materials

The following supporting information can be downloaded at: https://www.mdpi.com/article/10.3390/jof11110810/s1, S1: Accumulated amount of CO_2_ produced for *N. intermedia* monoculture, *R. oryzae* monoculture, *N. intermedia* and *R. oryzae* co-culture, sum of the nCO_2_ produced by the monocultures; S2: Luedeking–Piret–Monod script of modeling equations and model parameters for the *N. intermedia* and *R. oryzae* co-culture and their respective monocultures; S3: Script of the granola bar survey questions. References [52,72,73,74] are cited in the Supplementary Materials.

## Abbreviations

The following abbreviations are used in this manuscript:

AIL	Acid Insoluble lignin
ANOVA	Analysis of Variance
AP	Alternative protein
ASL	Acid soluble lignin
CER	CO_2_ evolution rate
DM	Dry matter
GRAS	Generally recognized as safe
IR	Inclusion rate
NF	Non-fermented
SSF	Solid-state fermentation
