# Supplementary material for: Upcycling Oat Hulls via Solid-State Fermentation Using Edible Filamentous Fungi: A Co-Culture Approach with Neurospora intermedia and Rhizopus oryzae
Source: J Fungi (Basel). 2025 Nov 14;11(11):810. doi: 10.3390/jof11110810 (PMC12653450; doi:10.3390/jof11110810)
Supplement: Supplementary file 1 [file jof-11-00810-s001.zip › jof-3942068-supplementary.pdf]

Supplementary Information
S1
Accumulated amount of CO_2 produced for *N. intermedia* monoculture, *R. oryzae* monoculture, *N. intermedia* and *R. oryzae* co-culture, sum of the nCO_2 produced by the monocultures

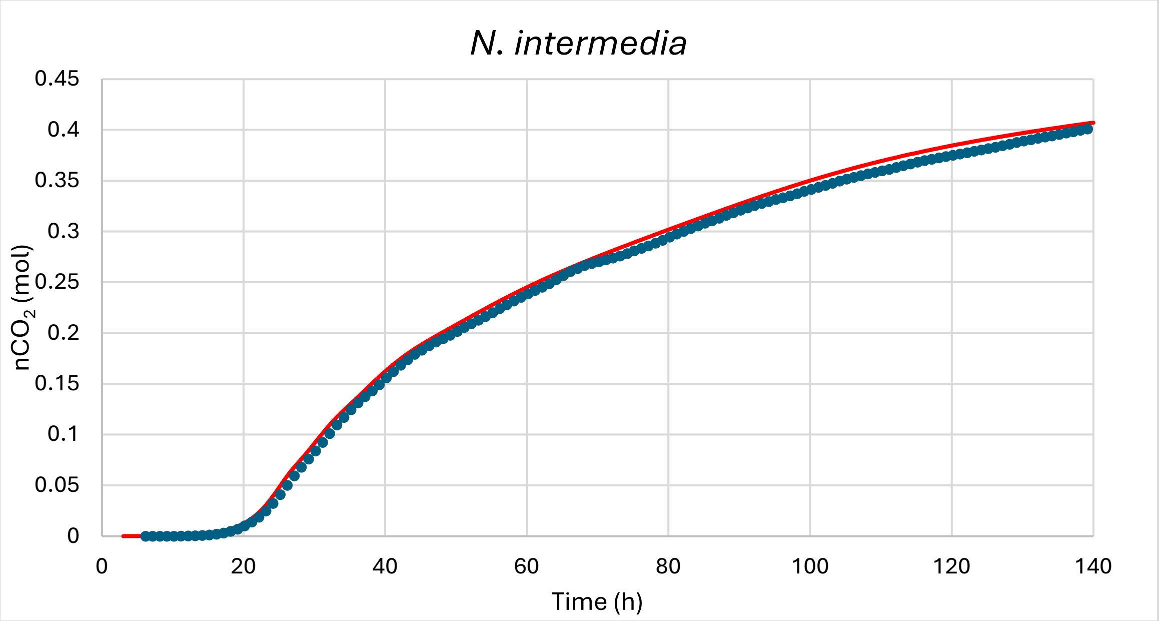

Measured (blue dots) and modeled (red curve) nCO_2 from *N. intermedia* monoculture.

Accumulated amount of CO_2: 0.39 moles

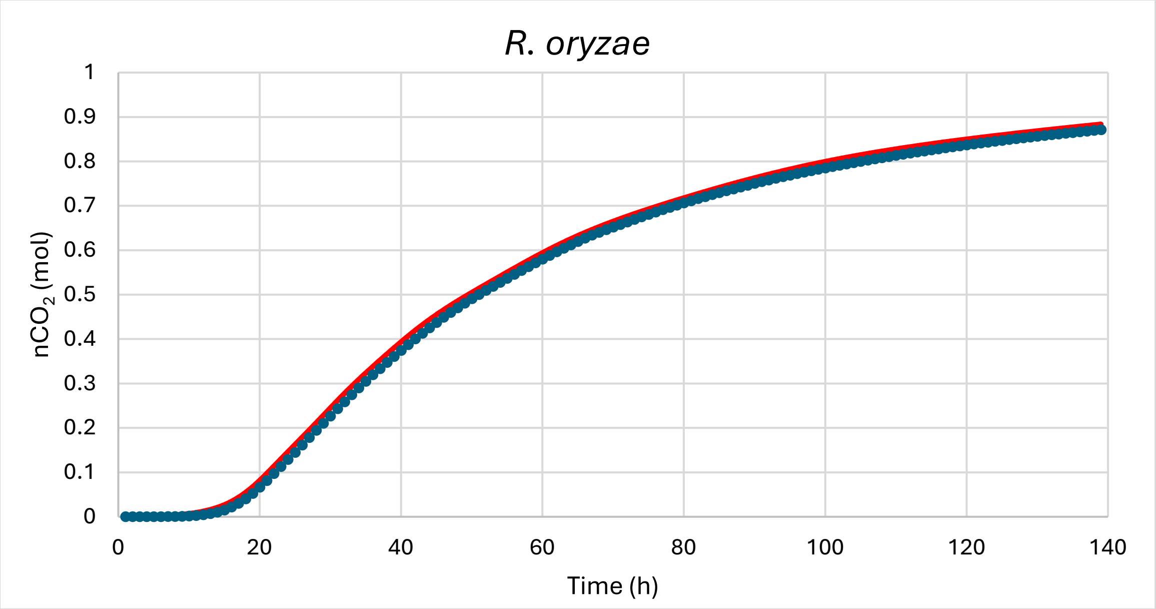

Measured (blue dots) and modeled (red curve) nCO_2 from *R. oryzae* monoculture.

Accumulated amount of CO_2: 0.84 moles

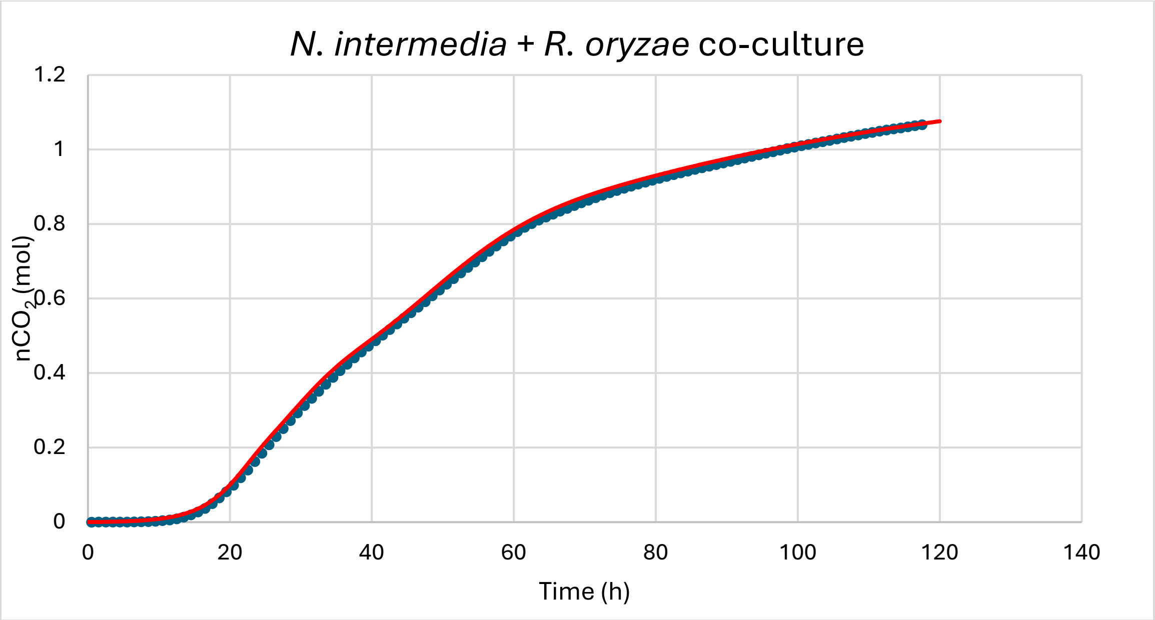

Measured (blue dots) and modeled (red curve) nCO_2 from *N. intermedia* and *R. oryzae* co-culture.

Accumulated amount of CO_2: 1.06 moles

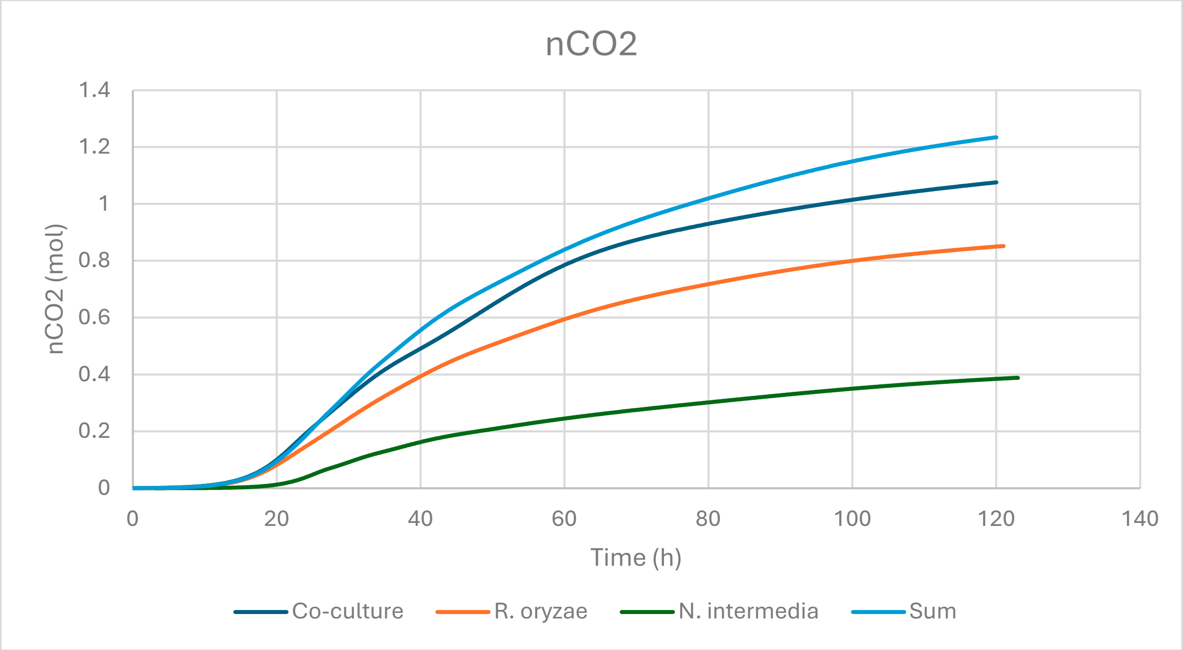

Comparison of model data: co-culture of R. oryzae and N. intermedia (dark blue); R. oryzae monoculture (orange); N. intermedia monoculture (green); sum of nCO_2 of both monocultures (light blue).

S2

Luedeking-Piret-Monod script of modeling equations and model parameters for the *N. intermedia* and *R. oryzae* co-culture and their respective monocultures.

To allow quantification and cross-fermentation comparisons of CO_2 evolution rates, CER from fungal solid-state fermentations, SSFs, we developed a model that describe the complex pattern of temporarily increasing and decreasing CER observed experimentally. The model assumes that carbon evolves from growth-associated metabolic processes as well as from maintenance metabolism, as described by the Leudeking-Piret model.

$$\mathrm{CER} = \alpha\mu X + \beta X \tag{s1}$$

where α is the growth-associated CO_2 evolution (unitless), μ the specific growth rate (h^{-1}), and β the CO_2 evolution stemming from maintenance (h^{-1}). X represents the amount of fungal biomass in terms of carbon moles (C-mol). When the model was executed, all variables and parameters were expressed in terms of carbon equivalents.

Specific growth rate

The model considers that carbon uptake in filamentous fungi is regulated, enabling the fungi to utilize different carbon sources sequentially, starting with the most preferable substrate supporting the highest specific growth rates (Kerkaert and Huberman 2023). The model included 5 substrates, each associated with a maximal specific growth rate, $\mu_{max,1}$ - $\mu_{max,5}$ (h^{-1}), where $\mu_{max,1} \geq \mu_{max,2} \geq \mu_{max,3} \geq \mu_{max,4} \geq \mu_{max,5}$. Specific growth rates on each of these substrates, μ_1 - μ_5 (h^{-1}) were calculated based on the Monod equation modified by an inhibition term, allowing more preferable substrates to inhibit the utilization of all the lesser preferable carbon sources.

$$\mu_1 = \frac{\mu_{max,1} \cdot S_1}{K_{S,1} + S_1} \tag{s2}$$

$$\mu_2 = \left(\frac{\mu_{max,2} \cdot S_2}{K_{S,2} + S_2}\right)\left(\frac{K_{I,1}}{S_1 + K_{I,1}}\right) \tag{s3}$$

$$\mu_3 = \left(\frac{\mu_{max,3} \cdot S_3}{K_{S,3} + S_3}\right)\left(\frac{K_{I,2}}{S_1 + S_2 + K_{I,2}}\right) \tag{s4}$$

$$\mu_4 = \left(\frac{\mu_{max,4} \cdot S_4}{K_{S,4} + S_4}\right)\left(\frac{K_{I,3}}{S_1 + S_2 + S_3 + K_{I,3}}\right) \tag{s5}$$

$$\mu_5 = \left(\frac{\mu_{max,5} \cdot S_5}{K_{S,5} + S_5}\right)\left(\frac{K_{I,4}}{S_1 + S_2 + S_3 + S_4 + K_{I,4}}\right) \tag{s6}$$

The symbols $S_1 - S_5$ represent the amount of substrate in the culture (C-mol), $K_{S,1} - K_{S,5}$ are half saturation (Monod) constants (C-mol), and $K_{S,1} - K_{S,4}$ are inhibition constants (C-mol).

Substrate utilization

Utilization rates of each of the 5 substrates is described by Equations s7 – s11.

$$\frac{dS_1}{dt} = \frac{1}{Y_{X/S1}} \frac{\mu_{max,1} \cdot S_1}{K_{S,1} + S_1} X \quad \text{(s7)}$$

$$\frac{dS_2}{dt} = \frac{1}{Y_{X/S2}} \left(\frac{\mu_{max,2} \cdot S_2}{K_{S,2} + S_2} \right) \left(\frac{K_{I1}}{S_1 + K_{I,1}} \right) X \quad \text{(s8)}$$

$$\frac{dS_3}{dt} = \frac{1}{Y_{X/S3}} \left(\frac{\mu_{max,3} \cdot S_3}{K_{S,3} + S_3} \right) \left(\frac{K_{I2}}{S_1 + S_2 + K_{I,2}} \right) X \quad \text{(s9)}$$

$$\frac{dS_4}{dt} = \frac{1}{Y_{X/S4}} \left(\frac{\mu_{max,4} \cdot S_4}{K_{S,4} + S_4} \right) \left(\frac{K_{I3}}{S_1 + S_2 + S_3 + K_{I,3}} \right) X \quad \text{(s10)}$$

$$\frac{dS_5}{dt} = \frac{1}{Y_{X/S5}} \left(\frac{\mu_{max,5} \cdot S_5}{K_{S,5} + S_5} \right) \left(\frac{K_{I4}}{S_1 + S_2 + S_3 + S_4 + K_{I,4}} \right) X \quad \text{(s11)}$$

where $Y_{X/S1}$ - $Y_{X/S5}$ are the yields of fungal biomass on each substrate (unitless).

Growth

Fungal growth on each of the 5 substrates is assumed to be additive and affected by maintenance.

$$\frac{dX}{dt} = Y_{X/S_1} \frac{dS_1}{dt} + Y_{X/S_2} \frac{dS_2}{dt} + Y_{X/S_3} \frac{dS_3}{dt} + Y_{X/S_4} \frac{dS_4}{dt} + Y_{X/S_5} \frac{dS_5}{dt} - \beta X \quad \text{(s12)}$$

Numerical modelling

In order to model the amounts of the different substrates present during the SSFs, Equations s2-s11 were solved numerically using Euler's method

$$S_{1,t+\Delta t} = S_{1,t} - \left(\frac{1}{Y_{X/S1}} \frac{\mu_{max,1} \cdot S_{1,t}}{K_{S,1} + S_{1,t}} X_t \right) \Delta t \quad \text{(s16)}$$

$$S_{2,t+\Delta t} = S_{2,t} - \left(\frac{1}{Y_{X/S2}} \left(\frac{\mu_{max,2} \cdot S_{2,t}}{K_{S,2} + S_{2,t}} \right) \left(\frac{K_{I,1}}{S_{1,t} + K_{I,1}} \right) X_t \right) \Delta t \quad \text{(s17)}$$

$$S_{3,t+\Delta t} = S_{3,t} - \left(\frac{1}{Y_{X/S3}} \left(\frac{\mu_{max,3} \cdot S_{3,t}}{K_{S,3} + S_3, t} \right) \left(\frac{K_{I,2}}{S_{1,t} + S_{2,t} + K_{I,2}} \right) X_t \right) \Delta t \quad \text{(s18)}$$

$$S_{4,t+\Delta t} = S_{4,t} - \left(\frac{1}{Y_{X/S4}} \left(\frac{\mu_{max,4} \cdot S_{4,t}}{K_{S,4} + S_4, t} \right) \left(\frac{K_{I,3}}{S_{1,t} + S_{2,t} + S_{3,t} + K_{I,3}} \right) X_t \right) \Delta t \quad \text{(s19)}$$

$$S_{5,t+\Delta t} = S_{5,t} - \left(\frac{1}{Y_{X/_{S5}}} \left(\frac{\mu_{max,5} \cdot S_{5,t}}{K_{S,5} + S_5, t} \right) \left(\frac{K_{I,4}}{S_{1,t} + S_{2,t} + S_{3,t} + S_{4,t} + K_{I,4}} \right) X_t \right) \Delta t \qquad \text{(s20)}$$

and the amount of fungal biomass was modelled using a numerical solution to Equation s12

$$\begin{aligned} X_{t+\Delta t} = X_t &+ Y_{X/_{S1}} (S_{1,t+\Delta t} - S_{1,t}) + Y_{X/_{S2}} (S_{2,t+\Delta t} - S_{2,t}) + Y_{X/_{S3}} (S_{3,t+\Delta t} - S_{3,t}) \\ &+ Y_{X/_{S4}} (S_{4,t+\Delta t} - S_{4,t}) + Y_{X/_{S5}} (S_{5,t+\Delta t} - S_{5,t}) - \beta X_t \Delta t \end{aligned} \qquad \text{(s21)}$$

at time intervals, $\Delta t = 0.01$ h^{-1}.

CO_2 evolution rate

The CER reflects the metabolic processes of fungal cultures (Vrabl et al. 2019). The growth-associated CER from the uptake of alle 5 carbon sources was assumed to be additive. Thus, Equation s1 becomes

$$\text{CER} = (\alpha_1 \mu_1 + \alpha_2 \mu_2 + \alpha_3 \mu_3 + \alpha_4 \mu_4 + \alpha_5 \mu_5) X + \beta X \qquad \text{(s22)}$$

where $\alpha_1 - \alpha_5$ represent the growth-associated CER's associated to each of the 5 carbon sources. Since the carbon assimilated by the fungi is either stored as biomass or released as CO_2

$$\alpha_i = 1 - Y_{X/Si} \qquad \text{(s23)}$$

where *i* denotes one specific carbon source. Equation s14 can be combined with Equation s13, yielding

$$\begin{aligned} \text{CER} = \Big(&\left(1 - Y_{X/_{S1}}\right) \mu_1 + \left(1 - Y_{X/_{S2}}\right) \mu_2 + \left(1 - Y_{X/_{S3}}\right) \mu_3 + \left(1 - Y_{X/_{S4}}\right) \mu_4 \\ &+ \left(1 - Y_{X/_{S5}}\right) \mu_5 + \beta \Big) X \end{aligned} \qquad \text{(s24)}$$

Cumulative CO_2 evolution

Finally, the cumulative amount of CO_2 released from the SSFs was calculated by summing the amount of CO_2 released in each time interval Δt

$$n_{CO2} = \sum_{t=0}^{t=j} CER_t \cdot \Delta t \qquad \text{(s25)}$$

where *j* denotes the number of time intervals since start.

Parameters

The different parameters were determined experimentally, selected based on literature, or estimated by fitting the modelled CER to the experimental CER. The model was fitted to data by eye. The specific growth rates on the most preferable carbon source, μ_1 was found experimentally, as described in Figure s1. Specific growth rates on the remainder carbon sources were estimated by fitting the model to data.

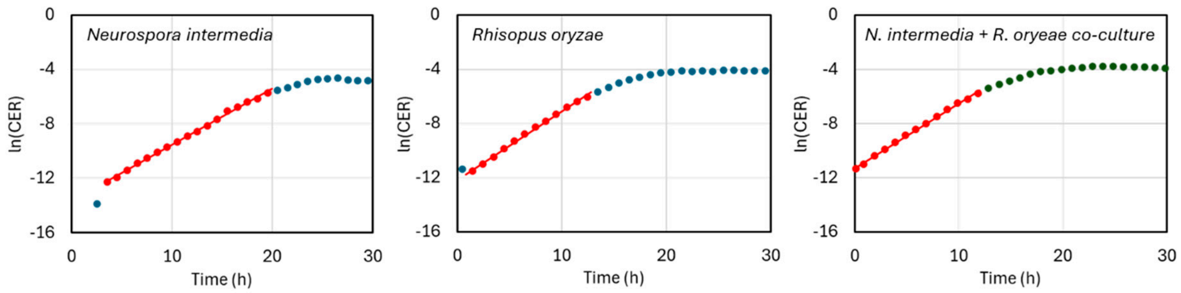

Figure s1. Plots of ln(CER) *vs*. time during the initial 30 h of SSF of oat hulls using *Neurospora intermedia*, *Rhizopus oryzae*, or co-culture of both species. Data from the initial phases, when CER increased exponentially indicated by red dots. Slopes of linear regression curves indicate μ_1 = 0.41, 0.51, and 0.42 h^{-1}, respectively.

The growth yields were taken to be 0.34 (based in carbon equivalents) on all carbon sources, which supposedly were mostly carbohydrates. This corresponds to a biomass yield of 0.42 based on weight (Larsen et al. 2004). The initial amounts of substrates, the initial amount of fungal biomass, the half saturation constants, and the inhibition coefficients were estimated by fitting the model to data. Modelling was initiated after initial lag-phases, t_{lag}, of 1 - 3 h. All parameters are listed in Table s1.

Table s1. Initial conditions and parameters used to model CER during SSF if oat hulls using *Neurospora intermedia*, *Rhizopus oryzae* or co-culture of both fungi. Values are calculated assuming 40% carbon in the substrates (common for carbohydrates) and 49% carbon in the fungal biomass (Roels 1980). Symbols are described in the text.

Parameter	unit	*N. intermedia*	*R. oryzae*	co-culture
Initial conditions				
X_0	g	0.002	0.02	0.03
$S_{1,0}$	g	6.5	12	25.5
$S_{2,0}$	g	8	25	30
$S_{3,0}$	g	12.5	28	58
$S_{4,0}$	g	8	22	10
$S_{5,0}$	g	11	14	5
t_{lag}	h	3	2	1
Growth and substrate utilization				
$\mu_{max,1}$	h^{-1}	0.41	0.53	0.42
$\mu_{max,2}$	h^{-1}	0.15	0.16	0.2
$\mu_{max,3}$	h^{-1}	0.1	0.1	0.1
$\mu_{max,4}$	h^{-1}	0.08	0.08	0.1
$\mu_{max,5}$	h^{-1}	0.08	0.08	0.08

$Y_{X/S1}$	-	0.34	0.34	0.34
$Y_{X/S2}$	-	0.34	0.34	0.34
$Y_{X/S3}$	-	0.34	0.34	0.34
$Y_{X/S4}$	-	0.34	0.34	0.34
$Y_{X/S5}$	-	0.34	0.34	0.34
$K_{S,1}$	g	2	12	18
$K_{S,2}$	g	2	13	38
$K_{S,3}$	g	10	30	100
$K_{S,4}$	g	20	50	100
$K_{S,5}$	g	50	100	100
$K_{I,1}$	g	1	8	4
$K_{I,2}$	g	0.4	2.5	4
$K_{I,3}$	g	0.3	1.5	1
$K_{I,4}$	g	0.3	1	1
CO_2 evolution				
α_1	-	0.66	0.66	0.66
α_2	-	0.66	0.66	0.66
α_3	-	0.66	0.66	0.66
α_4	-	0.66	0.66	0.66
α_5	-	0.66	0.66	0.66
β	h^{-1}	0.0019	0.0014	0.0015

References

Kerkaert JD and Huberman LB (2023) Regulation of nutrient utilization in filamentous fungi. Applied Microbiology and Biotechnology 107: 5873–5898. https://doi.org/10.1007/s00253-023-12680-4

Larsen B, Poulsen BR, Eriksen NT, Iversen JJL (2004) Homogeneous batch cultures of *Aspergillus oryzae* by elimination of wall growth in the Variomixing bioreactor. Applied Microbiology and Biotechnology 64: 192–198. https://doi.org/10.1007/s00253-003-1437-x

Roels JA (1980) Application of macroscopic principles to microbial metabolism. Biotechnology and Bioengineering 22: 2457–2514. https://doi.org/10.1002/bit.260221202

Vrabl P, Schinagl CW, Artmann DJ, Heiss B, Burgstaller W (2019) Fungal growth in batch culture – what we could benefit if we start looking closer. Frontiers in Microbiology 10: 2391. https://doi.org/10.3389/fmicb.2019.02391

S3
Script of the granola bar survey questions

For each variation (0 %, 15 % C, 20 % C, 20 % F, 25 % C):

1. Rate the appearance of this granola bar. (Scale: 1 – Very unattractive, 5 – Very attractive)
2. Rate the smell of this granola bar. (Scale: 1 – Very unpleasant, 5 – Very pleasant)
3. Rate the texture (mouthfeel). (Scale: 1 – Very unpleasant, 5 – Very pleasant)
4. Rate the flavor/taste. (Scale: 1 – Very unpleasant, 5 – Very pleasant)
5. Rate the overall acceptability of this granola bar. (Scale: 1 – Dislike very much, 5 – Like very much)
6. Which sample did you like the most? (Select one: 0 %, 15 % C, 20 %C, 20 % F, 25 % C)
7. Which sample did you like the least? (Select one: 0 %, 15 % C, 20 %C, 20 % F, 25 % C)
8. Would you consider buying a granola bar with fermented oat hulls if it offered additional health or environmental benefits? (Yes/No/Maybe)
